# Chinese Version of the EQ-5D Preference Weights: Applicability in a Chinese General Population

**DOI:** 10.1371/journal.pone.0164334

**Published:** 2016-10-06

**Authors:** Chunmei Wu, Yanhong Gong, Jiang Wu, Shengchao Zhang, Xiaoxv Yin, Xiaoxin Dong, Wenzhen Li, Shiyi Cao, Naomie Mkandawire, Zuxun Lu

**Affiliations:** 1 School of Public Health, Tongji Medical College, Huazhong University of Science and Technology, Wuhan, Hubei Province, China; 2 School of Basic Medicine, Gannan Medical University, Ganzhou, Jiangxi Province, China; 3 Xixiang Management Center of Community Health Service, Shenzhen, Guangdong Province, China; 4 Ningbo College of Health Sciences, Ningbo, Zhejiang Province, China; Zhejiang University, CHINA

## Abstract

**Objectives:**

This study aimed to test the reliability, validity and sensitivity of Chinese version of the EQ-5D preference weights in Chinese general people, examine the differences between the China value set and the UK, Japan and Korea value sets, and provide methods for evaluating and comparing the EQ-5D value sets of different countries.

**Methods:**

A random sample of 2984 community residents (15 years or older) were interviewed using a questionnaire including the EQ-5D scale. Level of agreement, convergent validity, known-groups validity and sensitivity of the EQ-5D China, United Kingdom (UK), Japan and Korea value sets were determined.

**Results:**

The mean EQ-5D index scores were significantly (*P*<0.05) different among the UK (0.964), Japan (0.981), Korea (0.987), and China (0.985) weights. High level of agreement (intraclass correlations coefficients > 0.75) and convergent validity (Pearson’s correlation coefficients > 0.95) were found between each paired schemes. The EQ-5D index scores discriminated equally well for the four versions between levels of 10 known-groups (*P*< 0.05). The effect size and the relative efficiency statistics showed that the China weights had better sensitivity.

**Conclusions:**

The China EQ-5D preference weights show equivalent psychometric properties with those from the UK, Japan and Korea weights while slightly more sensitive to known group differences than those from the Japan and Korea weights. Considering both psychometric and sociocultural issues, the China scheme should be a priority as an EQ-5D based measure of the health related quality of life in Chinese general population.

## Introduction

Health utilities (HUs) or health-state utility values (HSUVs) are key parameters for health economic studies[1]. In most cases, a health utility is generated using a preference based scoring algorithm of a generic existing instrument of health-related quality of life (HRQol). These algorithms are derived from the general population by a certain direct method. Internationally used instruments include the Quality of the Health Utilities Index (HUI)[2–4], the short form-36 health survey (SF-36)[5] and the EuroQol Group’s EQ-5D[6]. These instruments have been translated and tested in China and been used increasingly in different areas in the recent decades. However, no efforts were contributed to the development of value sets based on this population’s preference until 2014 a Chinese time trade-off (TTO) value set for the EQ-5D health states using a sample of the Chinese general population was published by Liu and his colleagues [7]. Actually, it is the first and only preference based on utility tariff of a HRQol instrument in mainland China.

The EQ-5D is one of the widely validated generic HRQol measures known as its simplicity. It contains a five-dimension descriptive system (i.e., mobility, self-care, usual activities, pain/discomfort and anxiety/depression) and a visual analogue scale (VAS). All the dimensions are grouped into three levels (i.e., no problem, some problem and extreme problem), thus one person’s health status was described as one of the 243 (3^5^) theoretical possible health states by the EQ-5D classification system and a VAS score between 0 and 100. Each dimension would be divided into five levels for the EQ-5D-5L scale, but the EQ-5D usually means the EQ-5D-3L scale if not specially stated. In 1993, Dolan et al. carried out the Measurement and Valuation of Health (MVH) study and modeled the world’s first country-specific population-based preference scoring algorithm for converting an EQ-5D health state to a utility score in the United Kingdom (UK)[8]. Researchers have been adapting the methodology used in the MVH study and applying to generate local value sets in a number of countries and areas[9–14].

The EQ-5D is also commonly used as a measure of health status in China. It has been recommended by *China Guidelines for Pharmacoeconomic Evaluations 2011* for a measure for HRQol and health utility. The National Health Services Survey (NHSS) in China in 2008 have established the Chinese norm of the EQ-5D. Results showed the HRQoL valuations in Chinese population aged 15 years and above and could be used for international comparisons. However, the valuations were limited to itemized descriptions[15], as no China value set had been established for the EQ-5D. To date, the EQ-5D UK and Japan weights have been used in most studies conducted in China while whether these weights are applicable in the Chinese general population has hardly been validated.

The concern would be no longer a trouble with introducing a properly built and validated China EQ-5D value set. The econometric model constructed from the valuation study by Liu et al. yielded a good model fit[7]. However, the reliability, validity and sensitivity of the China value set has not been evaluated after established and whether it is applicable for Chinese population has not been confirmed. In addition, due to drawbacks in the study design, the sample was not nationally representative and could lead to bias in predicting the health preference of Chinese population. In order to examine whether the China EQ-5Dvalue set would be applicable for the Chinese general population, this study tested its psychometric properties and the differences from three value sets: the UK[8], Japan[9] and South Korea schemes[13] using a general population from China. The UK and Japan schemes were usually used in China before with little evidence about the applicability used in the Chinese general population. The Korea scheme was selected to compare with the China scheme as the social, economical and cultural backgrounds were much similar between Korea and China, which would make it valuable to compare the schemes of the two countries. This study provided an alternative way for further studies to evaluate and characterize differences of the EQ-5D country-specific value sets.

## Materials and Methods

### Subjects and procedures

A survey was conducted in Xixiang Street, Bao’an District of Shenzhen in southeast China from October to November, 2013. There were over 600 thousand people living in Xixiang Street and about 80% were from other areas of China. The survey was a part of a large study, which aimed to provide information for community diagnosis for local health sectors. The whole protocol and the questionnaire were adapted from that of the NHSS 2013 and had been reviewed by relative experts. Xixiang Street had 33 communities. In each community, 40 families were designed to be randomly selected, representing 1320 families in total. Face-to-face household interviews were conducted by trained local interviewers using a questionnaire to all family members. One of the parents or another adult family member living together answered the questionnaire on behalf of the minor participant aged five years or below or those who could hardly answer the questionnaire themselves. The main contents of the questionnaire included the socio-economic characteristics, health status (including the Simplified Chinese version of the EQ-5D-3L scale), health risk factors and health service needs and utilization. Like the NHSS protocol, the EQ-5D scale was only interviewed among participants aged 15 years and older, as the Chinese child-friendly version of the EQ-5D (EQ-5D-Y) for younger respondents was not available. Physical examinations were also conducted among participants aged 15 years and older in local community health service centers to measure blood pressure, height, weight, waistline and hipline. In order to present the final results as a Chinese population value set, the study also reported results which applied corrective weights to reflect the Chinese national age/sex distribution in 2013. Participant information was anonymised prior to analysis. All participants provided written consent to participate in this study. Written consent of minor participants was provided by their parents or other adult next of kin, caretakers, or guardians who lived together. The study protocol including consent procedure was approved by the Institutional Review Board of Tongji Medical College, Huazhong University of Science and Technology, Wuhan, China.

### EQ-5D: China, UK, Japan and Korea preference weights

The EQ-5D China, UK, Japan and Korea preference weights were compared in the study. Before the China weights were established, the UK and Japan weights were usually used in China. The Korea weights were also compared considering the similar social, economical and cultural backgrounds between Korea and China, which would make the comparison valuable.

The UK N3 model for EQ-5D preference weights was derived from the MVH study by Dolan based on a representative sample of non-institutionalized adult population in England, Scotland and Wales in 1993[8]. As the first and well validated EQ-5D value set in the world, the UK scheme is widely used when there are not local ones offered or for comparison with other schemes in sensitivity analysis. The Japan valuation study was conducted based on the quasi MVH protocol by collecting a random sample of adult population from three prefectures in 1998[9]. A plain main effects model for the Japan social value set was established with high goodness of fit. The Japan value set is the first EQ-5D tariff in Asia. Considering similar culture contexts, it has often been used in Asian countries where no local tariffs were available. There are two EQ-5D value sets for South Korea[12–13]. Lee et al. carried out a study using instruments and protocol similar to the MVH protocol based on a nationally representative sample in 2006 and established a model with promoted goodness-of-fit than the former one[13]. Liu et al. developed an EQ-5D value set for the Chinese general population in 2011 based on the Paris protocol[7] which was revised from the MVH protocol by Kind[14].

### Data analysis

Respondents aged 15 years and above were included in this study. The EQ-5D index scores for each respondent were calculated using the UK, Japan, South Korea and China algorithms. The differences of scores generated from the four value sets were compared using ANOVA followed by post-hoc Bonferroni tests.

The four preference weights were compared with regard to the criteria of three psychometric properties: level of agreement (i.e. intra-observational reliability), convergent validity and known-groups validity and sensitivity. In order to examine whether the newly established China value set is applicable for the Chinese national general population, the results reported in this study applied corrective weights to reflect the Chinese national age/sex distribution in 2013.

#### Level of agreement

The agreements among the EQ-5D scores using the UK, US, and Japan preference weights were assessed using intraclass correlations coefficients (ICCs) and two-way random-effects model with absolute agreement and Bland-Altman agreement plots. According to the Rosner’s criteria, ICC below 0.40 is regarded as poor agreement, 0.40–0.75 as fair to good agreement, and 0.75 and above as excellent agreement[16].

#### Convergent validity

Convergent validity of the four weighting schemes was evaluated by assuming that subjects with high scores from China EQ-5D scheme had high scores from other EQ-5D schemes and the EQ-VAS scores, and high global ratings of health status. The global rating is from the first item of SF-36 scale with five response options: excellent, very good, good, fair, and poor[5]. Pearson’s correlation coefficient was used to describe correlations between two schemes of the EQ-5D index scores as well as between the EQ-5D index scores and the VAS scores. Spearman’s rho correlation coefficient was used to describe correlations between the EQ-5D index scores and the global well-being ratings. The strength of the correlation was defined that strong correlations were > 0.50, moderate correlations ranged between 0.35 and 0.49 and weak correlations ranged between 0.20 and 0.34[17].

#### Known-groups validity

Known-groups validity of the EQ-5D preference weights was analyzed by making assumptions that the EQ-5D weights have the ability to discriminate subjects from different socio-economic, risk factor related and health status known groups. Previous studies had shown different distributions of EQ-5D index scores according to the respondents’ demographic, socio-economic status (SES), and other health related indicators[12, 18, 19]. This study examined whether the EQ-5D index scores were significantly different among subgroups by age, gender, education, income, employment status, health status, and health service utilization. Education, income and employment status were the SES indicators most commonly used. The indicators for health status included four variables: VAS score, global rating, chronic condition diagnosed and/or treated during the past 6 months, and onset of diseases or injuries during the last two weeks. Outpatient visit during the last two weeks and hospitalization during the past 12 months were indicators for health service utilization.

The educational level was classified into below primary school, primary school, junior middle school, senior middle school, college and above. The income was defined as the household annual income divided by the numbers of persons living in the family within the last half-year. Respondents were divided into five income groups of equal size: the lowest income group had an income below 15,000 RMB; the second group from 15,000 to 23,333 RMB; the third group from 23,334 to 29,999 RMB; the fourth group from 30,000 to 49,999 RMB; the fifth and highest income group 50,000 RMB and above. Employment status was classified into employed, unemployed, student and retired. Global rating was defined as mentioned in the section of convergent validity. Respondents were categorized into two groups by with or without a chronic disease, disease/injury during the last two weeks, outpatient visit during the last two weeks, hospitalization during the past 12 months, as well as having VAS scores below versus equal or above the median, respectively.

To identify the differences of EQ-5D index scores among known groups, independent t-test and ANOVA analysis were used. When the respondents were divided by a dichotomous variable, independent t-test was used to identify statistically significant effects on utility scores of different groups, while ANOVA test was used for a polytomous variable. The differences of EQ-5D index scores among known groups were compared with the minimal important difference (MID) to find if they were meaningful. There was no authoritative recommendation for the MID of EQ-5D index scores. Compared with those of other preference-based health-related quality of life instruments, the MID of the EQ-5D would be larger[20–21]. A change of 0.05 was considered to be a MID as this is equivalent to the mean change in the SF-36 (0.03) and the HUI (0.04 for HUI2 and 0.07 for HUI3)[21].

#### Sensitivity

The sensitivity of the EQ-5D weighting schemes was estimated by assessing the Cohen’s d effect size (ES) statistic and the relative efficiency (RE) statistic. ES is defined as the differences between known groups divided by the standard deviation (SD) of EQ-5D index scores. The equation is as follows:

$$\text{Cohen }d\text{=}\frac{{\bar{x}}_{1}-{\bar{x}}_{2}}{s_{\text{pooled}}}$$

Cohen suggests that an ES ≥ 0.2 would be a meaningful difference, and 0.2 ≤ ES < 0.5 indicates a “small” difference, 0.5 ≤ ES < 0.8 indicates a “moderate” difference, and ES ≥ 0.8 indicates a “large” difference[22]. RE is based on the ratio of the F or squared t statistic for one algorithm divided by that for another algorithm. RE ≥ 1 means the efficiency of the numerator is better or equal than that of the denominator and vice versa[23]. All statistical analyses were performed using SPSS17.0 (IBM Corp., Armonk, NY, USA).

## Results

### Characteristics of respondents

A total of 1320 families were selected and 63 families refused to the survey, with a respondent rate of 95.2%. There were 4148 respondents interviewed. Twenty-five respondents were excluded for missing data on age (17), gender (6) and both age and gender (2), remaining 4123. Among them, 3028 respondents were 15 years old and above. Forty-four were excluded for missing data regarding to the EQ-5D five-dimensional system. Finally, 2984 respondents were included in our analysis, with an average age of 36.7 (SD = 12.4) years. Detailed results are shown in Table 1.

**Table 1:** Characteristics of the respondents (n = 2984).

Characteristic		Value
Sex	Female	50.3%
Age (year)	Mean ± SD	36.7 ± 12.4
Race	Han	96.6%
Marital status		
Married		86.8%
Unmarried		11.4%
Others		1.6%
Education		
Below primary		2.0%
Primary		10.0%
Junior high		30.4%
Senior high and its equivalent		30.7%
College and above		26.7%
Income (¥)	Mean ± SD	115878±135273
EQ-5D VAS score	Mean ± SD	84.3±10.8
Global rating		
Excellent		9.9%
Very good		44.3%
Good		31.4%
Fair		11.8%
Poor		0.9%
Six-month chronic condition		14.7%
Two-week disease/injury		5.5%
Two-week outpatient visit		3.6%
One-year hospitalization		7.8%

### Quality profile of the EQ-5D

The results showed that 7.0% of the respondents reported some or extreme problem on one or more EQ-5D dimensions, with most respondents reporting problems with the pain/discomfort dimension and least reporting problems with the self-care dimension. The ratio rose to 11.2% in the weighted analysis (S1 Table). S2 Table shows the weighted percentages of respondents with some or extreme problem in each dimension by age groups and gender. In general, the percentages of problems increased in older age groups and were higher in women than men in each EQ-5D dimension in most age groups.

Table 2 shows the distribution of the EQ-5D index scores and ceiling effect of the respondents. There were 93.0% of the respondents who reported with no problems in any dimension. The percentages of ceiling effects were higher in men (94.6%) than in women (91.4%). The China weights (mean ± SD: 0.985 ± 0.059) generated slightly higher mean utility scores compared to the UK (mean ± SD: 0.964 ± 0.133) and Japan (0.981 ± 0.073) weights, and slightly lower mean utilities compared to the Korea weights (0.987 ± 0.053). All score means were significantly different between two weights (*P* < 0.05) but less than MID, ranging from 0.002 (Korea/China) to 0.022 (Korea/UK). Distributions were skewed to the left for all weights.

**Table 2:** Distribution of EQ-5D index scores using China, UK, Japan and Korea weights.

Weights	Min	Mean	SD	Median	IQR	Ceiling effect
*Overall*						93.0%
China	0.249	0.985	0.059	1.000	0.000	
UK	-0.264	0.964	0.133	1.000	0.000	
Japan	0.134	0.981	0.073	1.000	0.000	
Korea	0.138	0.987	0.053	1.000	0.000	
*Male*						94.6%
China	0.249	0.988	0.055	1.000	0.000	
UK	-0.264	0.972	0.120	1.000	0.000	
Japan	0.134	0.985	0.066	1.000	0.000	
Korea	0.195	0.990	0.049	1.000	0.000	
*Female*						91.4%
China	0.292	0.982	0.063	1.000	0.000	
UK	0.028	0.957	0.144	1.000	0.000	
Japan	0.195	0.977	0.078	1.000	0.000	
Korea	0.138	0.984	0.057	1.000	0.000	
*15–44 yrs*						95.4%
China	0.483	0.991	0.045	1.000	0.000	
UK	0.028	0.977	0.107	1.000	0.000	
Japan	0.430	0.988	0.057	1.000	0.000	
Korea	0.482	0.992	0.040	1.000	0.000	
*45–64 yrs*						89.2%
China	0.483	0.979	0.068	1.000	0.000	
UK	0.193	0.946	0.158	1.000	0.000	
Japan	0.532	0.972	0.084	1.000	0.000	
Korea	0.627	0.981	0.057	1.000	0.000	
*65+ yrs*						62.6%
China	0.249	0.905	0.151	1.000	0.153	
UK	-0.264	0.795	0.283	1.000	0.473	
Japan	0.134	0.885	0.170	1.000	0.232	
Korea	0.138	0.916	0.145	1.000	0.137	

All means were significantly different between two weights (*P* < 0.05).

### Level of agreement

Table 3 shows the agreements among the EQ-5D scores valuated by the four preference weights using ICCs with two-way random-effects model with absolute agreement. Based on Rosner’s criterion, all the ICCs were very high and excellent agreement was found between any two of the four weights. The highest ICC of 0.987 was between the China and Korea weights, and the lowest between the UK and Korea weights (0.780). After truncation of EQ-5D scores below 0 for sensitivity analysis, the ICCs remained the same between the pairs of Asian weights and decreased by less than 0.01 between the UK and any Asian weights.

**Table 3:** Pearson correlation coefficients and ICCs between China, UK, Japan and Korea weights.

Preference weights	Correlation coefficient^a^	ICC (95% CI)	ICC^b^ (95% CI)
China/UK	0.962	0.824 (0.790–0.851)	0.820 (0.786–0.848)
China /Japan	0.980	0.979 (0.975–0.982)	0.979 (0.975–0.982)
China /Korea	0.981	0.987 (0.986–0.988)	0.987 (0.986–0.988)
UK / Japan	0.990	0.903 (0.882–0.919)	0.901 (0.880–0.917)
UK/ Korea	0.950	0.780 (0.739–0.812)	0.773 (0.731–0.806)
Japan / Korea	0.982	0.965 (0.957–0.970)	0.965 (0.957–0.970)

ICC: intraclass correlation coefficient.

^a^All *P* values < 0.001.

^b^ICC with truncation of EQ-5D scores < 0 for sensitivity analysis.

**Fig 1:**
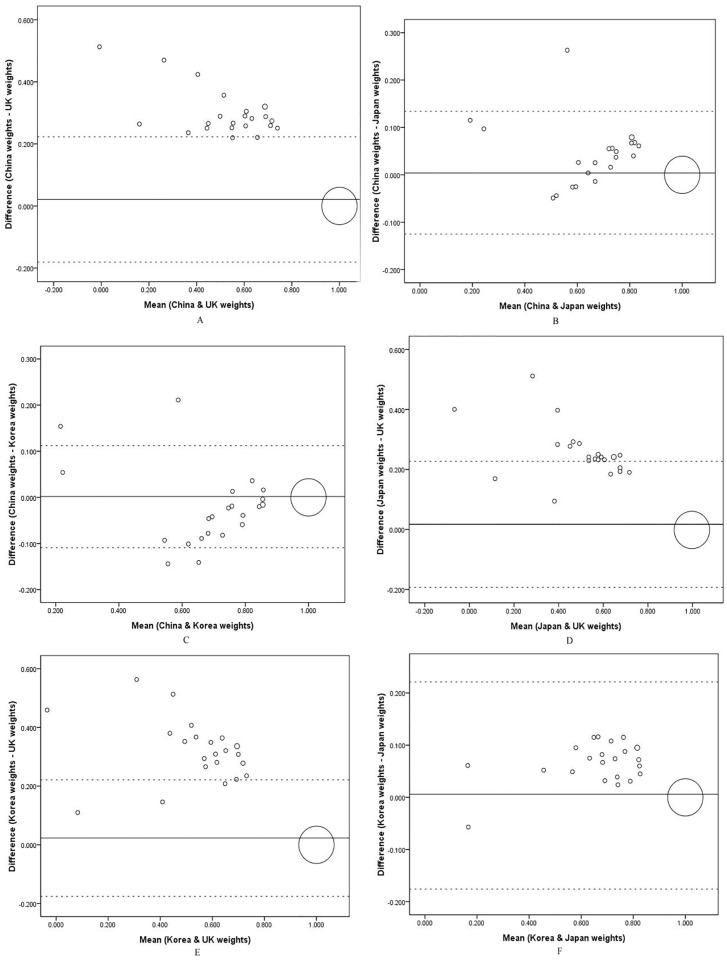
a-f. The Bland-Altman plots of EQ-5D scores derived from the China, UK, Japan, and Korea preference weights.

Fig 1a–1f showed the Bland-Altman plots of the pairs of the four weighted EQ-5D scores to compare the degree of agreement. The mean of the differences (*d*) and the limits of agreement (95% CI of *d*) were indicated by lines. Perfect agreement was found between each pair of the weights as over 90% of the difference scores were in the limits of agreement.

### Convergent validity

The Pearson correlation coefficients of EQ-5D index scores for the pairs of weights were all above 0.95 (*P* < 0.001) (Table 3). The correlations between the EQ-5D utilities and the EQ-VAS scores were significant (*P* < 0.001), with 0.352 for the China and Japan weights, 0.353 for the UK weights, and 0.343 for the Korea weights, respectively. Spearman's rho correlation coefficients were -0.194 between all the EQ-5D based scores and the global ratings of health status (*P* < 0.001).

### Known-groups validity

Overall, the known-groups validity and sensitivity measures were close for the EQ-5D scores generated by the four preference weights (Table 4). The EQ-5D scores derived from each value set were significantly different for all the 11 categories of known groups (*P* < 0.01). When the respondents were divided indicators of age, global rating, and outpatient visit respectively, the score differences by each of the four preference weights were larger than MID. The score differences larger than MID were also observed between respondents with education level of below primary school and college and above using China, UK and Japan weights, with and without chronic diseases and disease/injury in the last two weeks using UK weights, and with and without disease/injury in the last two weeks using Japan weights.

**Table 4:** Known-groups validity of the EQ-5D index scores using China, UK, Japan and Korea weights.

Group	China	UK	Japan	Korea
Male*	0.988	0.972	0.985	0.990
Female	0.982	0.957	0.977	0.984
15–44 yrs**	0.991	0.977	0.988	0.992
45–64 yrs	0.979	0.946	0.972	0.981
≥ 65 yrs	0.905^#^	0.795^#^	0.885^#^	0.916^#^
College and above**	0.992	0.979	0.989	0.993
Senior high and its equivalent	0.987	0.967	0.982	0.988
Junior high	0.988	0.970	0.985	0.990
Primary	0.965	0.919	0.956	0.968
Below primary	0.941^#^	0.866^#^	0.928^#^	0.949
Lowest income**	0.981	0.954	0.976	0.984
Low income	0.975	0.944	0.970	0.979
Middle income	0.989	0.971	0.985	0.990
High income	0.990	0.973	0.986	0.990
Highest income	0.991	0.975	0.987	0.992
Employed**	0.991	0.977	0.988	0.992
Student	1.000	1.000	1.000	1.000
Retired	0.968	0.912	0.955	0.972
Unemployed	0.963	0.919^#^	0.955	0.967
Chronic condition -**	0.990	0.975	0.987	0.991
Chronic condition +	0.954	0.888^#^	0.940	0.959
Disease/injury -**	0.988	0.970	0.984	0.989
Disease/injury +	0.943	0.870^#^	0.929^#^	0.948
Outpatient visit -**	0.988	0.969	0.984	0.989
Outpatient visit +	0.927^#^	0.838^#^	0.911^#^	0.934^#^
Hospitalization -*	0.988	0.969	0.984	0.989
Hospitalization +	0.967	0.925	0.959	0.970
Global rating excellent**	0.996	0.989	0.994	0.996
Global rating very good	0.993	0.983	0.991	0.994
Global rating good	0.985	0.965	0.981	0.987
Global rating fair	0.954	0.886	0.940	0.959
Global rating poor	0.889^#^	0.756^#^	0.868^#^	0.908^#^
EQ-5D VAS score ≥ median**	0.996	0.990	0.995	0.996
EQ-5D VAS score < median	0.974	0.937	0.967	0.977

*: *P* < 0.01;

**: *P* < 0.001;

^#^: Mean difference between known groups of each weights > minimal important difference (0.05).

### Sensitivity

Among nine of the 11 known groups, the estimates of ES for all schemes were larger than 0.2, presenting a certain degree of sensitivity. Large differences with the ES ≥ 0.8 were observed for four known groups, i.e. age, education level, outpatient visit and global rating (Table 5). There were moderate differences with the ES between 0.5 and 0.8 for groups divided by chronic condition and two-week morbidity for all schemes. Small differences existed for two known groups divided by history of hospitalization and VAS levels.

**Table 5:** Sensitivity of the EQ-5D index scores using China, UK, Japan and Korea weights.

Group	China	UK	Japan	Korea
Gender* (male vs female)				
ES	0.101	0.117	0.112	0.103
RE	1.000	1.326	1.220	1.043
Age* (15–44 vs ≥ 65 yrs)				
ES	1.579	1.518	1.545	1.538
RE	1.000	0.920	0.964	0.984
Education* (college and higher vs below primary)				
ES	1.017	0.972	0.994	1.027
RE	1.000	0.967	0.984	0.968
Income* (lowest vs highest group)				
ES	0.173	0.158	0.163	0.170
RE	1.000	0.796	0.857	0.873
Employment status* (employed vs unempolyed)				
ES	0.203	0.221	0.215	0.203
RE	1.000	1.028	1.009	1.004
Chronic condition* (yes vs no)				
ES	0.617	0.672	0.663	0.629
RE	1.000	1.243	1.154	0.958
Disease/injury * (yes vs no)				
ES	0.777	0.763	0.767	0.787
RE	1.000	1.257	1.135	0.906
Outpatient visit * (yes vs no)				
ES	1.040	1.005	1.019	1.066
RE	1.000	1.282	1.139	0.891
Hospitalization * (yes vs no)				
ES	0.354	0.339	0.348	0.355
RE	1.000	1.106	1.057	0.914
Global rating* (excellent vs poor)				
ES	2.252	2.164	2.194	2.180
RE	1.000	1.124	1.075	0.942
VAS score* (≥ median vs < median)				
ES	0.371	0.404	0.389	0.356
RE	1.000	1.184	1.098	0.924

ES: effect size; RE: relative efficiency; vs: versus.

*: The EQ-5D index scores of different subgroups were significantly different using the same weights (*P* < 0.05).

Comparing the four preference weights, the China schemes generated the largest RE estimates for three variables: age, education, and household income. The UK weighted scores were most efficient at discriminating the differences between groups for the rest eight known-groups with the biggest REs. The UK weights also provided slightly larger ES values than the three other weights in groups divided by three variables. Besides, the ES estimates from the China weights were the largest in groups related to three variables and the second largest in groups related to four variables as well.

## Discussion

The study examined the psychometric properties of the China preference weights for EQ-5D utility scores in a general community population. The overall results showed that there were small differences and similar validity of the China, Japan, Korea and UK EQ-5D preference weights as a measure of HRQol in the Chinese general population. The scores generated by the four weights were close with mean differences of statistical but meaningless significance. Excellent agreement and a strong correlation were found between any two of the four weights as well. Besides, the four weights discriminated similarly well among demographic, SES, and health status known groups. The China value set showed weak superiority of discriminative ability for age, education, and household income subgroups. As far as we could comprehend, this was the first time for the China preference weights to be tested. The study provided evidence about validity and performance of the UK, Japan and Korea EQ-5D preference weights in the Chinese general population, which was rarely reported.

Limited studies had compared preference values and their algorithms for EQ-5D health states in different countries. However, strong positive correlations[9, 13, 24–26], high level of agreement[24, 25], and similar validities[26, 27] with mixed results of REs [24–26, 28–30] among the UK, Japan and Korea value sets had been reported in previous studies in general and patient samples from and outside of these countries, including China[9, 13, 24–27]. In particular, Chang et al. [27] found that in a representative Taiwan sample the UK and Japan utilities could discriminate equally among the known groups of five-level global health status, two-week outpatient visits, and one-year hospitalization, as in the present study. Meaningless differences in absolute magnitude among the EQ-5D’s utilities from the three value sets were also reported in previous studies both in the general population[24, 26, 27] and patients[25, 28].

On the other hand, significantly different scores existed in this study and some previous studies comparing the UK, Japan and other national tariffs[24, 27]. The cross-country discrepancies may ascribe to actual differences in peoples’ preferences for health after ruling out effects of technical aspects from the development process of value sets: noise introduced during the translation process of the EQ-5D scale and the valuation procedure (such as TTO), as well as differences in the study design and method[9–10]. Four studies used TTO as the valuation procedure. Considering the simplicity of the EQ-5D scale and TTO, the effects of the former two factors were unlikely to be great. Referred to the third factor, three protocols were used in the four studies: the MVH protocol, a modified version of the MVH protocol and the Paris protocol. The latter two protocols were all based on the MVH protocol and used in developing the EQ-5D value sets around the world. The four studies also equally developed acceptable models using the three protocols. Then people’s preferences for health would be the main source of differences.

Inter-country comparisons of the EQ-5D country-specific models revealed that there was a significant tendency that Asian raters gave greater weights to the functional dimensions of health whereas Western raters gave greater weights to the dimensions of pain/discomfort and anxiety/depression[7–13]. And valuations for EQ-5D health status in the generic public were broadly similar across Western countries[18, 31–32], while valuations across Asian countries like Japan and China are consistent in which the percentages of respondents reporting problems in each dimension were very low except for the pain/discomfort dimension[15, 19, 33]. Similar findings were also achieved in international comparisons of the SF-6D[34–39]. Moreover, our results also suggested that the three Asian weights performed closer to each other than to the UK weights in many ways. These observations illustrated similarities in health preferences among people in China, Korea and Japan.

The choice of weighting scheme in health valuations was a subject of debate[40]. It was clear that the China scheme established a model of perfect goodness-of-fit with estimates superior to the UK and Korea models and close to the Japan model[7–9, 13]. The general criteria underlying the priority of weighting schemes was that, in some parts due to the cultural influences on subject ratings and differences in study settings, health valuations will perform differently when applied to different populations[41]. Culture was a complex issues, but previous studies and this study implied that Asian value systems perform closer to each other and better than the UK and US systems in the Asians[9, 13, 29, 32, 35], as referred above. Besides, the protocols for model estimation studies were similar but there were as much as 97 health states directly valued in the China study, which therefore minimized the interpolation spaces in the model estimation. Most importantly, the China version of the EQ-5D preference weights was found in our study to discriminate known-groups efficiently and even a little better than the Japan and Korea weights considering both ES and RE estimates. Based on the above factors, the China EQ-5D preference weights should be used preferentially for Chinese population.

For studies where the EQ-5D value sets were evaluated and compared[24–27, 41], the present study had a relative large sample. However, due to limited resource restraints, it was carried out in only one city, and such findings should be interpreted carefully to the national population as the sample were not nationally representative. To make the conclusion generalized, Shenzhen was selected as high proportion of the people were from other areas around China, thus the population in the study could represent the national population in a certain degree. In addition, the findings of the present study were similar to those of recent studies comparing the psychometric properties of the EQ-5D value sets of other countries in Chinese population, for instance, one study in an urban community population of northern China[24] and another in a rural community population of southern China[26]. It suggested that age, sex and location would not affect the validity as evidence from previous studies had showed[9, 10, 41, 42]. In this regard, the results of this study were reliable and could be able to be generalized to the national population in a certain degree. There was another limitation of the study that the EQ-5D is usually self administered but was interviewed face-to-face in this study like in the NHSS to making it possible to collect information from those with reading difficulties. In order to reduce inter-cluster correlation and interview bias, the interviewers were trained to avoid interference between family members during investigation. Finally, the study was based on a cross-sectional investigation and other measurement properties such as test-retest reliability and responsiveness were not available. They should be addressed for further examination.

## Conclusions

In conclusion, the validity and sensitivity of the China EQ-5D value set is verified in the Chinese general population by comparing with those of the UK, Japan, and Korea. The China TTO value set for the EQ-5D should be given preference for use for the general adult Chinese population and an increasingly wide utilization of the EQ-5D scale should be encouraged in China in the future.

## Supporting Information

S1 File Chinese Version of the EQ-5D Preference Weights—Data. (XLSX)

S1 Table Percentages of respondents reporting problems on each EQ-5D dimension. (DOCX)

S2 Table Weighted percentages of respondents reporting moderate and severe problems on each EQ-5D dimension by age group and sex (%). (DOCX)

## References

[1] Torrance GW. Measurement of health state utilities for economic appraisal: a review. J Health Econ. 1986;5(1)1–30. 10.1016/0167-6296(86)90020-2 10311607

[2] Torrance GW, Boyle MH, Horwood SP. Application of multi-attribute utility theory to measure social preferences for health states. Oper Res. 1982;30(6):1043–69. 10.1287/opre.30.6.1043 10259643

[3] Torrance GW, Feeny DH, Furlong WJ, Barr RD, Zhang Y, Wang Q. Multiattribute utility function for a comprehensive health status classification system. Health Utilities Index Mark 2. Med Care. 1996;34(7):702–22. 8676608 10.1097/00005650-199607000-00004

[4] Feeny DH, Furlong WJ, Torrance GW, Goldsmith CH, Zhu Z, DePauw S, et al Multiattribute and single-attribute utility functions for the Health Utilities Index Mark 3 system. Med Care. 2002;40:113–28. 10.1097/00005650-200202000-00006 11802084

[5] Ware JE, Sherbourne CD. The MOS 36-Item Short-Form Health Survey (SF-36): Conceptual framework and item selection. Med Care 1992;30:473–83. 10.1097/00005650-199206000-00002 1593914

[6] Group EuroQol. EuroQol: a new facility for the measurement of health related quality of life. Health Policy. 1990;16(3):199–208. 10109801 10.1016/0168-8510(90)90421-9

[7] Liu GG, Wu H, Li M, Gao C, Luo N. Chinese time trade-off values for EQ-5D health states. Value Health. 2014;17(5):597–604. 10.1016/j.jval.2014.05.007 25128053

[8] Dolan P. Modeling valuations for EuroQol health states. Med Care. 1997;35(11):1095–108. 10.1097/00005650-199711000-00002 9366889

[9] Tsuchiya A, Ikeda S, Ikegami N, Nishimura S, Sakai I, Fukuda T, et al Estimating an EQ-5D population value set: the case of Japan. Health Econ. 2002; 11(4):341–353. 10.1002/hec.673 12007165

[10] Greiner W, Claes C, Busschbach JJ, von der Schulenburg JM. Validating the EQ-5D with time trade off for the German population. Eur J Health Econ. 2005;6(2):124–30. 10.1007/s10198-004-0264-z 19787848

[11] Shaw JW, Johnson JA, Coons SJ. US valuation of the EQ-5D health states: development and testing of the D1 valuation model. Med Care. 2005;43(3):203–20. 10.1097/00005650-200503000-00003 15725977

[12] Jo MW, Yun SC, Lee SI. Estimating quality weights for EQ-5D health states with the time trade-off method in South Korea. Value Health. 2008; 112(11): 1186–9. 10.1111/j.1524-4733.2008.00348.x 18489498

[13] Lee YK, Nam HS, Chuang LH, Kim KY, Yang HK, Kwon IS, et al South Korean time trade-off values for EQ-5D health states: modeling with observed values for 101 health states. Value Health. 2009;12(8):1187–93. 10.1111/j.1524-4733.2009.00579.x 19659703

[14] Kind P. A revised protocol for the valuation of health states defined by the EQ-5D-3L classification system: learning the lessons from the MVH study. York, UK: Centre for Health Economics, University of York, 2009.

[15] Sun S, Chen J, Johannesson M, et al Population health status in China EQ-5D results, by age, sex and socio-economic status, from the National Health Services Survey 2008. Qual Life Res. 2011;20(3):309–20. 10.1007/s11136-010-9762-x 21042861 PMC3052443

[16] Rosner B. Fundamental of Biostatistics. Pacific Grove, California: Duxbury Thomson Learning; 2000.

[17] Colton T. Regression and correlation In Statistics in medicine Little, Brown and Company; 1974:307–10.

[18] Kind P, Dolan P, Gudex C, Williams A. Variations in population health status: results from a United Kingdom national questionnaire survey. BMJ. 1998;316(7133):736–41. 10.1136/bmj.316.7133.736 9529408 PMC28477

[19] Wang H, Kindig DA, Mullahy J. Variation in Chinese population health related quality of life: results from a EuroQol study in Beijing, China. Qual Life Res. 2005;14(1):119–32. 10.1007/s11136-004-0612-6 15789946

[20] Walters SJ, Brazier JE. Comparison of the minimally important difference for two health state utility measures: EQ-5D and SF-6D. Qual Life Res. 2005;14(6):1523–32. 10.1007/s11136-004-7713-0 16110932

[21] Marra CA, Woolcott JC, Kopec JA, Shojania K, Offer R, Brazier JE, et al A comparison of generic, indirect utility measures (the HUI2, HUI3, SF-6D, and the EQ-5D) and disease-specific instruments (the RAQoL and the HAQ) in rheumatoid arthritis. Soc Sci Med. 2005;60(7):1571–82. 10.1016/j.socscimed.2004.08.034 15652688

[22] Cohen J. A power primer. Psychol Bull. 1992;112(1):155–9. 10.1037/0033-2909.112.1.155 19565683

[23] Hays RD, Anderson R, Revicki D. Psychometric considerations in evaluating health-related quality of life measures. Qual Life Res. 1993 12;2(6):441–9. 10.1007/BF00422218 8161978

[24] Wu YQ, Liu K, Tang X, Cao Y, Wang JW, Li N, et al Empirical research of measuring elderly health utility in the outskirts of Beijing by using European quality of life 5-dimensions. Beijing Da Xue Xue Bao. 2012;44(3):397–402. Chinese 22692310

[25] Sakthong P, Charoenvisuthiwongs R, Shabunthom R. A comparison of EQ-5D index scores using the UK, US, and Japan preference weights in a Thai sample with type 2 diabetes. Health Qual Life Outcomes. 2008;6:71 10.1186/1477-7525-6-71 18811935 PMC2559828

[26] Jin H, Wang B, Gao Q, Chao J, Wang S, Tian L, et al Comparison between EQ-5D and SF-6D Utility in Rural Residents of Jiangsu Province, China. PLoS One. 2012;7(7):e41550 10.1371/journal.pone.0041550 22848526 PMC3407238

[27] Chang TJ, Tarn YH, Hsieh CL, Liou WS, Shaw JW, Chiou XG. Taiwanese version of the EQ-5D: validation in a representative sample of the Taiwanese population. J Formos Med Assoc. 2007;106(12):1023–31. 10.1016/S0929-6646(08)60078-9 18194908

[28] Kim J, Kwak HW, Lee WK, Kim HK. Impact of photodynamic therapy on quality of life of patients with age-related macular degeneration in Korea. Jpn J Ophthalmol. 2010;54(4):325–30. 10.1007/s10384-010-0825-x 20700801

[29] Pickard AS, Ray S, Ganguli A, Cella D. Comparison of FACT- and EQ-5D-based utility scores in cancer. Value Health. 2012;15(2):305–11. 10.1016/j.jval.2011.11.029 22433762

[30] Busschbach J, Weijnen T, Nieuwenhuizen M, Oppe S, Badia X, Dolan P, et al A comparison of EQ-5D time trade-off values obtained in Germany, the United Kingdom and Spain. The Measurement and Valuation of Health Status Using EQ-5D: A European Perspective. 2003;143–65. 10.1007/978-94-017-0233-1_9

[31] Johnson JA, Coons SJ. Comparison of the EQ-5D and SF-12 in an adult US sample. Qual Life Res. 1998;7(2):155–66. 9523497 10.1023/a:1008809610703

[32] Greiner W, Weijnen T, Nieuwenhuizen M, Oppe S, Badia X, Busschbach J, et al A single European currency for EQ-5D health states. Results from a six-country study. Eur J Health Econ. 2003;4(3):222–31. 10.1007/s10198-003-0182-5 15609189

[33] Ikeda S, Ikegami N. Health Status in Japanese Population: Results from Japanese EuroQol Study. Med Soc. 1999;9(3): 83–91. Japanese 10.4091/iken1991.9.3_83

[34] Fukuhara S, Suzukamo Y. Manual of SF-36v2 Japanese version. Institute for Health Outcomes & Process Evaluation Research, Kyoto, Japan (2004).

[35] Brazier J, Roberts J, Deverill M. The estimation of a preference-based measure of health from the SF-36. J Health Econ. 2002;21(2):271–92. 10.1016/S0167-6296(01)00130-8 11939242

[36] Li L, Wang HM, Shen Y. Chinese SF-36 Health Survey: translation, cultural adaptation, validation, and normalization. J Epidemiol Community Health. 2003;57(4):259–63. 10.1136/jech.57.4.259 12646540 PMC1732425

[37] Jenkinson C, Coulter A, Wright L. Short Form 36 (SF-36) health survey questionnaire: normative data for adults of working age. BMJ. 1993;306(6890):1437–40. 8518639 10.1136/bmj.306.6890.1437 PMC1677870

[38] Fukuhara S, Suzukamo Y. Manual of SF-36v2 Japanese version. Institute for Health Outcomes & Process Evaluation Research, Kyoto, Japan (2004).

[39] Cruz LN, Fleck MP, Oliveira MR, Camey SA, Hoffmann JF, Bagattini AM, et al Health-related quality of life in Brazil: normative data for the SF-36 in a general population sample in the south of the country. Cien Saude Colet. 2013;18(7):1911–21. 10.1590/S1413-81232013000700006 23827895

[40] Prieto L, Sacristán JA. What is the value of social values? The uselessness of assessing health-related quality of life through preference measures. BMC Med Res Methodol. 2004;4:10 10.1186/1471-2288-4-10 15117417 PMC415546

[41] Huang IC, Willke RJ, Atkinson MJ, Lenderking WR, Frangakis C, Wu AW. US and UK versions of the EQ-5D preference weights: does choice of preference weights make a difference? Qual Life Res. 2007;16(6):1065–72. 10.1007/s11136-007-9206-4 17415683

[42] Wu J, Han Y, Zhao FL, Zhou J, Chen Z, Sun H. Validation and comparison of EuroQoL-5 dimension (EQ-5D) and Short Form-6 dimension (SF-6D) among stable angina patients. Health Qual Life Outcomes. 2014;25(12):156 10.1186/s12955-014-0156-6 25343944 PMC4213514

