# Supplementary material for: Chinese Version of the EQ-5D Preference Weights: Applicability in a Chinese General Population
Source: PLoS One. 2016 Oct 6;11(10):e0164334. doi: 10.1371/journal.pone.0164334 (PMC5053600; doi:10.1371/journal.pone.0164334)
Supplement: S1 Table — (DOCX) [file pone.0164334.s002.docx]

Chinese version of the EQ-5D preference weights: Applicability in a Chinese general population

| S1 Table. Percentages of respondents reporting problems on each EQ-5D dimension | | | | | | | | |
| --- | --- | --- | --- | --- | --- | --- | --- | --- |
| Sample Source | N | EQ-5D VAS (0-100) | EQ-5D dimensions (% with any problems by dimension) | | | | | |
|  |  | VAS score | Mobility | Self-care | Usual activities | Pain/ discomfort | Anxiety/ depression | Any dimension |
| UK (age≥18) | 3395 | 82.5 | 18.4 | 4.2 | 16.3 | 33.0 | 20.9 | 43.1 |
| US* (age≥18) | 427 | 82.2 | 14.0 | 3.0 | 14.0 | 40.0 | 24.0 |  |
| Canada (age≥18) | 1518 | 78.7 | 22.2 | 4.0 | 19.1 | 43.6 | 28.6 | 53.0 |
| Japan (age≥18) | 621 | 77.8 | 7.2 | 1.8 | 5.2 | 20.0 | 8.5 | 25.0 |
| Spain (age≥18) | 12245 | 71.1 | 11.2 | 2.0 | 6.9 | 26.3 | 12.5 | 33.0 |
| Beijing (age≥12) | 2991 | 77.0 | 4.9 | 2.0 | 3.3 | 18.0 | 6.1 | 22.4 |
|  |  |  | (3.4) | (1.4) | (2.2) | (13.0) | (5.3) | (17.2) |
| NHSS 2008(age≥15) | 120703 | 80.1 | 4.9 | 3.1 | 4.6 | 8.9 | 5.8 |  |
| Shenzhen (age≥15) | 2984 | 84.3 | 1.4 | 0.7 | 1.0 | 5.5 | 2.1 | 7.0 |
|  | (3387) | (82.4) | (3.2) | (1.9) | (2.7) | (9.0) | (2.4) | (11.2) |

Weighted estimations are in the parentheses.

UK: Kind P et al. 1998. US: Johnson JA et al. 1998. Canada: Johnson JA et al. 2000. Spain: Badia X et al. 1998. Beijing: Wang H et al. 2005. NHSS: Sun S et al. 2011.

*Since the authors did not provide the original data, the numbers are roughly estimated from figures in their paper.
