# Supplementary material for: Chinese Version of the EQ-5D Preference Weights: Applicability in a Chinese General Population
Source: PLoS One. 2016 Oct 6;11(10):e0164334. doi: 10.1371/journal.pone.0164334 (PMC5053600; doi:10.1371/journal.pone.0164334)
Supplement: S2 Table — (DOCX) [file pone.0164334.s003.docx]

Chinese version of the EQ-5D preference weights: Applicability in a Chinese general population

| S2 Table. Weighted percentages of respondents reporting moderate and severe problems on each EQ-5D dimension by age group and sex (%) | | | | | | | | | | | | | | | | | | | | | | | | | | | | | | | | | | | |
| --- | --- | --- | --- | --- | --- | --- | --- | --- | --- | --- | --- | --- | --- | --- | --- | --- | --- | --- | --- | --- | --- | --- | --- | --- | --- | --- | --- | --- | --- | --- | --- | --- | --- | --- | --- |
| Age (years) | 15-19 | 20-24 | | 25-29 | | 30-34 | | 35-39 | | 40-44 | | 45-49 | | 50-54 | | 55-59 | | 60-64 | | 65-69 | | 70-74 | | 75-79 | | 80-84 | | 85+ | | Total | | |  |  |  |
| Male |  |  | |  | |  | |  | |  | |  | |  | |  | |  | |  | |  | |  | |  | |  | |  | | |  |  |  |
| *Mobility* |  |  | |  | |  | |  | |  | |  | |  | |  | |  | |  | |  | |  | |  | |  | |  | | |  |  |  |
| some | 0.0 | 0.0 | | 0.0 | | 0.7 | | 0.4 | | 0.5 | | 0.0 | | 4.4 | | 3.9 | | 0.0 | | 6.9 | | 5.0 | | 25.0 | | 0.0 | | 0.0 | | 0.9 | | |  |  |  |
| extreme | 0.0 | 0.0 | | 0.0 | | 0.0 | | 0.4 | | 0.0 | | 0.0 | | 0.0 | | 0.0 | | 0.0 | | 0.0 | | 0.0 | | 25.0 | | 0.0 | | 0.0 | | 0.1 | | |  |  |  |
| *self-care* |  |  | |  | |  | |  | |  | |  | |  | |  | |  | |  | |  | |  | |  | |  | |  | | |  |  |  |
| some | 0.0 | 0.8 | | 0.5 | | 0.3 | | 0.4 | | 0.5 | | 0.0 | | 1.5 | | 2.0 | | 0.0 | | 3.4 | | 0.0 | | 25.0 | | 0.0 | | 0.0 | | 0.6 | | |  |  |  |
| extreme | 0.0 | 0.0 | | 0.0 | | 0.0 | | 0.0 | | 0.0 | | 0.0 | | 0.0 | | 0.0 | | 0.0 | | 0.0 | | 0.0 | | 25.0 | | 0.0 | | 0.0 | | 0.1 | | |  |  |  |
| *usual activities* | | |  | |  | |  | |  | |  | |  | |  | |  | |  | |  | |  | |  | |  | |  | |  | | |  |  |
| some | 0.0 | 0.0 | | 0.0 | | 0.7 | | 0.4 | | 0.5 | | 0.0 | | 1.5 | | 2.0 | | 0.0 | | 6.9 | | 5.0 | | 25.0 | | 0.0 | | 0.0 | | 0.7 | | |  |  |  |
| extreme | 0.0 | 0.0 | | 0.0 | | 0.0 | | 0.0 | | 0.0 | | 0.0 | | 0.0 | | 0.0 | | 0.0 | | 0.0 | | 0.0 | | 0.0 | | 0.0 | | 0.0 | | 0.0 | | |  |  |  |
| *pain/discomfort* | | |  | |  | |  | |  | |  | |  | |  | |  | |  | |  | |  | |  | |  | |  | |  | | |  |  |
| some | 0.0 | 0.8 | | 0.0 | | 2.0 | | 3.8 | | 6.0 | | 0.0 | | 10.3 | | 13.7 | | 10.5 | | 20.7 | | 25.0 | | 25.0 | | 25.0 | | 0.0 | | 4.0 | | |  |  |  |
| extreme | 0.0 | 0.0 | | 0.0 | | 0.0 | | 0.0 | | 0.5 | | 0.0 | | 0.0 | | 0.0 | | 0.0 | | 0.0 | | 0.0 | | 25.0 | | 0.0 | | 0.0 | | 0.1 | | |  |  |  |
| *anxiety/depression* |  |  | |  | |  | |  | |  | |  | |  | |  | |  | |  | |  | |  | |  | |  | |  | | |  |  |  |
| some | 0.0 | 0.8 | | 1.8 | | 1.7 | | 2.1 | | 4.0 | | 0.0 | | 1.5 | | 2.0 | | 0.0 | | 6.9 | | 5.0 | | 0.0 | | 0.0 | | 0.0 | | 1.9 | | |  |  |  |
| extreme | 0.0 | 0.0 | | 0.0 | | 0.0 | | 0.0 | | 0.5 | | 0.0 | | 0.0 | | 0.0 | | 0.0 | | 0.0 | | 0.0 | | 0.0 | | 0.0 | | 0.0 | | 0.1 | | |  |  |  |
| Female |  |  | |  | |  | |  | |  | |  | |  | |  | |  | |  | |  | |  | |  | |  | |  | | |  |  |  |
| *mobility* |  |  | |  | |  | |  | |  | |  | |  | |  | |  | |  | |  | |  | |  | |  | |  | | |  |  |  |
| some | 1.4 | 1.4 | | 0.7 | | 0.0 | | 0.6 | | 1.3 | | 3.4 | | 1.4 | | 4.4 | | 3.6 | | 11.1 | | 16.7 | | 20.0 | | 66.7 | | 0.0 | | 1.7 | | |  |  |  |
| extreme | 0.0 | 0.0 | | 0.0 | | 0.0 | | 0.6 | | 0.0 | | 0.0 | | 0.0 | | 0.0 | | 0.0 | | 0.0 | | 0.0 | | 0.0 | | 0.0 | | 50.0 | | 0.1 | | |  |  |  |
| *self-care* |  |  | |  | |  | |  | |  | |  | |  | |  | |  | |  | |  | |  | |  | |  | |  | | |  |  |  |
| some | 0.0 | 0.7 | | 0.3 | | 0.0 | | 0.0 | | 0.0 | | 0.0 | | 0.0 | | 0.0 | | 3.6 | | 3.7 | | 16.7 | | 20.0 | | 33.3 | | 0.0 | | 0.6 | | |  |  |  |
| extreme | 0.0 | 0.0 | | 0.0 | | 0.0 | | 0.0 | | 0.0 | | 0.0 | | 0.0 | | 0.0 | | 0.0 | | 0.0 | | 0.0 | | 0.0 | | 0.0 | | 50.0 | | 0.1 | | |  |  |  |
| *usual activities* | | |  | |  | |  | |  | |  | |  | |  | |  | |  | |  | |  | |  | |  | |  | | |  | | |  |
| some | 1.4 | 0.7 | | 0.3 | | 0.0 | | 0.6 | | 0.6 | | 0.8 | | 0.0 | | 2.2 | | 5.5 | | 7.4 | | 16.7 | | 40.0 | | 66.7 | | | 0.0 | | | 1.3 | | |  |
| extreme | 0.0 | 0.0 | | 0.0 | | 0.0 | | 0.0 | | 0.0 | | 0.0 | | 0.0 | | 0.0 | | 0.0 | | 0.0 | | 0.0 | | 0.0 | | 0.0 | | | 50.0 | | | 0.1 | | |  |
| *pain/discomfort* | | |  | |  | |  | |  | |  | |  | |  | |  | |  | |  | |  | |  | |  | |  | | |  | | |  |
| some | 2.9 | 1.4 | | 3.1 | | 4.0 | | 4.0 | | 7.5 | | 11.9 | | 10.8 | | 6.7 | | 20.0 | | 33.3 | | 41.7 | | 60.0 | | 66.7 | | | 0.0 | | | 6.7 | | |  |
| extreme | 0.0 | 0.0 | | 0.0 | | 0.0 | | 0.0 | | 0.6 | | 0.0 | | 0.0 | | 0.0 | | 1.8 | | 0.0 | | 0.0 | | 0.0 | | 0.0 | | | 0.0 | | | 0.1 | | |  |
| *anxiety/depression* | | |  | |  | |  | |  | |  | |  | |  | |  | |  | |  | |  | |  | |  | |  | | |  | | |  |
| some | 0.0 | 1.4 | | 1.4 | | 2.5 | | 1.7 | | 1.9 | | 4.2 | | 0.0 | | 4.4 | | 3.6 | | 7.4 | | 0.0 | | 8.3 | | 33.3 | | | 0.0 | | | 2.2 | | |  |
| extreme | 0.0 | 0.0 | | 0.0 | | 0.0 | | 0.0 | | 0.6 | | 0.0 | | 0.0 | | 0.0 | | 0.0 | | 0.0 | | 0.0 | | 0.0 | | 0.0 | | | 0.0 | | | 0.1 | | |  |
